# Implementation and assessment of a prevention with positives intervention among people living with HIV at five hospitals in Thailand

**DOI:** 10.1371/journal.pone.0170558

**Published:** 2017-02-03

**Authors:** Benjamas Baipluthong, Thanomsak Anekthananon, Warangkana Munsakul, Supunnee Jirajariyavej, Suvanna Asavapiriyanont, Ubonsri Hancharoenkit, Anuvat Roongpisuthipong, Sarika Pattanasin, Michael Martin, Lisa Guntamala, Rangsima Lolekha

**Affiliations:** 1 Division of Global HIV and TB, U.S. Centers for Disease Control and Prevention, Nonthaburi, Thailand; 2 Faculty of Medicine, Siriraj Hospital, Mahidol University, Bangkok, Thailand; 3 Faculty of Medicine, Vajira Hospital, Navamindharadhiraj University, Bangkok, Thailand; 4 HIV Clinic, Taksin Hospital, Bangkok, Thailand; 5 Obstetrics and Gynecology Unit, Rajavithi Hospital, Bangkok, Thailand; 6 HIV Clinic, Wiang Pa Pao Hospital, Chiang Rai, Thailand; 7 Bureau of AIDS, TB and STIs, Department of Disease Control, Thailand Ministry of Public Health, Nonthaburi, Thailand; Azienda Ospedaliera Universitaria di Perugia, ITALY

## Abstract

**Background:**

We implemented a hospital-based prevention with positives (PwP) intervention among people living with HIV (PLHIV) that included HIV transmission risk screening, short HIV prevention messages, family planning, HIV disclosure counseling, and partner HIV testing at five hospitals in Thailand. We assessed changes in sexual risk behaviors among PLHIV who received the PwP services at the hospitals.

**Methods:**

From January 2008-March 2009, we systematically selected a subset of PLHIV receiving care at the five hospitals to offer participation in the PwP intervention. We collected demographic, risk behavior, and laboratory data using a standardized questionnaire. We analyzed data from PLHIV who completed at least four visits, using generalized estimating equations to identify baseline participant characteristics that were associated with adopting sexual practices less likely to be associated with HIV transmission during follow-up.

**Results:**

A total of 830 PLHIV were interviewed and 756 (91.1%) completed four visits. The median age of these 756 participants was 37 years, 400 (52.9%) were women, and 475 (62.8%) had a steady partner. At baseline, 353 (74.3%) of the steady partners had been tested for HIV and 132 (37.4%) had tested negative. Among the 756 PLHIV, 427 (56.5%) reported having sex in the 3 months before enrollment and 413 (54.6%) in the 3 months before the fourth visit. The proportion reporting having vaginal or anal sex without a condom decreased from 20.8% at baseline to 5.1% at the fourth visit (p<0.001). Factors associated (p<0.05) with abstinence or 100% condom use at follow-up visits included: completing ≥ two visits, being diagnosed with HIV for longer than 3 months, and receiving HIV prevention messages from a doctor (versus a nurse or counselor).

**Conclusion:**

Safe sex behaviors increased among PLHIV receiving PwP services, suggesting that expansion of hospital-based PwP services may reduce the number of new HIV infections in Thailand.

## Introduction

In 2014, the Government of Thailand issued new HIV treatment guidelines [1] recommending antiretroviral treatment (ART) for people living with HIV (PLHIV) regardless of CD4 count. ART was recommended for all PLHIV because of new evidence showing that early initiation of ART can improve the health of PLHIV, suppress HIV viral load, and reduce the risk of transmitting HIV to others [2–4]. The early use of ART provides PLHIV another tool to reduce the risk that they will transmit HIV to their sexual and needle-sharing partners. However, although the risk of HIV transmission with consistent ART use is low, it is not zero [5] and data from the HIV treatment and care cascades in many countries [6, 7] indicate that a substantial number of PLHIV who need ART are not receiving ART and that less than 50% of PLHIV engaged in care achieve undetectable HIV viral loads. Thus, behavioral and other risk-reduction interventions such as *Prevention with Positives* (PwP) remain important tools to prevent HIV transmission.

PwP interventions encourage PLHIV to take steps to maintain and improve their health, to prevent transmission of HIV and other sexually transmitted infections (STIs) to sexual and needle-sharing partners, and to prevent unintended pregnancies to limit HIV transmission from HIV-infected mothers to their new-born infants [8] while allowing full and satisfying emotional and sexual relationships [8–12]. A recent meta-analysis of 21 randomized clinical trials showed that PwP interventions, including either group or individual interventions, significantly reduced the frequency of condomless anal and vaginal intercourse [13].

**Fig 1:**
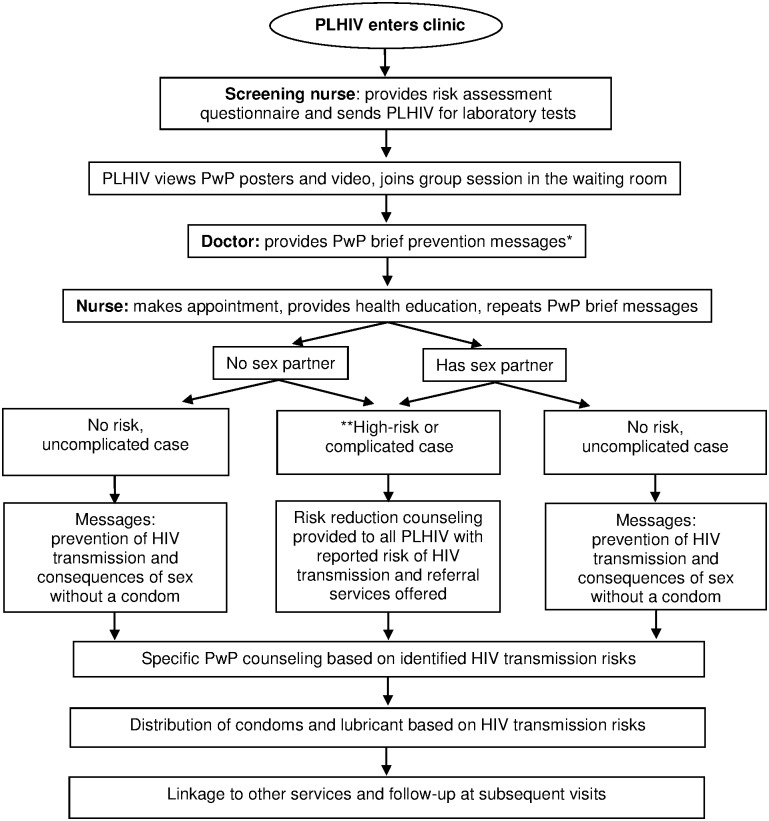
Flowchart of the Prevention with Positives (PwP) program for people living with HIV (PLHIV) in Thai hospitals. *Short prevention messages are provided to all PLHIV at routine clinical visits, covering all strategies in the initial visit and specific patient-centered strategies at follow-up visits. **High-risk/complicated case defined as people who inject drugs who report sharing needles, people who report sex without a condom, and people who have a history of STIs.

Division of Global HIV and TB (DGHT), U.S. Centers for Disease Control and Prevention (CDC), Thailand office worked with Thailand MOPH to develop a PwP model for health care settings that included a package of HIV prevention tools and services for PLHIV and their partners (Fig 1). The model was piloted in seven settings in Thailand during 2008–2009 [8, 14]. We previously described the PwP model and use of dual contraceptive methods by PLHIV who participated in the PwP program [8]. Here we briefly describe the PwP model, assess the risk behaviors of PLHIV who received PwP services and completed four clinical visits, and examine factors associated with a change to sexual behaviors less likely to be associated with HIV transmission.

## Materials and methods

### The Prevention with Positives (PwP) model

Staff from the Thailand MOPH, Bureau of AIDS, Tuberculosis, and STIs and DGHT worked with health care providers from seven hospitals (i.e., three government (MOPH) hospitals, two Bangkok Metropolitan Administration (BMA) hospitals, one MOPH STI clinic, and one university hospital) to develop PwP tools and determine what services to include in the PwP model using published PwP models to guide discussions [8, 15–17]. The goals of the PwP intervention were to prevent HIV transmission by screening PLHIV for behavior risks, provide targeted risk reduction messages, improve PLHIV ART adherence, screen PLHIV for and treat STIs, promote HIV status disclosure to partners, and encourage partner HIV testing and family planning [8, 18].

The PwP tools and services in the preliminary PwP package included a self-administered risk assessment questionnaire, educational posters, scripts for brief provider-delivered prevention messages, an educational flipchart, a participant information pamphlet, and guidance for client-centered counseling [14]. These materials were field tested at the seven pilot sites and the package was revised and finalized based on feedback from field site staff. The risk assessment questionnaire included questions about participant’s current ART regimen and dosing frequency, ART adherence (i.e., the number of doses missed in the previous 3 days and the number of doses missed in the previous month), sexual activity in the previous 3 months, condom use during the most recent sexual exposure, alcohol consumption during the previous 3 months, STI symptoms, partner HIV disclosure status, HIV testing history, and use of family planning tools. At each clinical visit, PLHIV were given the risk assessment questionnaire to complete. Trained health care staff and PLHIV volunteers were available to assist participants with the questionnaire as needed. Health care providers in the seven sites were trained to deliver the PwP package and to provide the brief prevention messages to all PLHIV attending the clinic based on risks reported in the risk assessment questionnaire. PLHIV who injected drugs, reported having sex without a condom, were sex workers, and had a history of STIs were referred for additional, more specific, participant-centered counseling (e.g., risk reduction counseling, partner HIV disclosure counseling, ART adherence counseling). When indicated, participants were referred for other services (e.g., antenatal care or peer support groups). The study protocol and consent forms were approved by the Ethical Review Committees of the Thailand MOPH, the BMA, Siriraj Hospital, and the U.S. CDC Institutional Review Board. PLHIV who signed the consent form were enrolled in the PwP program.

### Data collection and analysis

Non-pregnant PLHIV 18 years old and older who received services at the seven hospitals from January 2008 to March 2009 were eligible to enroll. At each site, every sixth eligible person was provided information about the PwP program and the assessment by the trained counselors. PLHIV who signed the consent form were enrolled in the assessment. Participants completed a baseline questionnaire and were followed every three months until they completed three follow-up visits for a total of four visits. If an individual did not agree to enroll, the next eligible person was provided information about the assessment and invited to enroll.

Among the seven sites, one site, an out-patient STI clinic providing diagnostic and treatment services for STIs and HIV testing, did not provide follow-up data and one hospital was unable to provide sufficient data for analysis. These two sites were excluded from this analysis. The five hospitals included in the analysis were Rajavithi Hospital, Siriraj Hospital, Taksin Hospital, Vajira Hospital, and Wiang Pa Pao Hospital (Table 1).

**Table 1:** Characteristics of hospitals participating in prevention with positives intervention assessment, Thailand, 2008–2009.

Site	Hospital description	Managing Institution	Approximate number of PLHIV receiving care during the study	Location
Rajavithi Hospital	Tertiary care hospital (1,182 in-patient beds)	Thailand Ministry of Public Health	1,000	Bangkok
Siriraj Hospital	Tertiary care, university hospital (2,221 in-patient beds)	Mahidol University	1,200	Bangkok
Taksin Hospital	Tertiary care hospital (450 in-patient beds)	Bangkok Metropolitan Administration	1,000	Bangkok
Vajira Hospital	Tertiary care, university hospital (875 in-patient beds)	Bangkok Metropolitan Administration	1,000	Bangkok
Wiang Pa Pao Hospital	Community hospital (60 in-patient beds)	Thailand Ministry of Public Health	500	Chiang Rai

Data were entered into a Microsoft Access database and analyzed using STATA (version 11.0, Stata Corp., Texas, United States). In Thailand in 2008–2009, people receiving ART were followed at hospitals or clinics every two to three months. Only data from participants who completed four or more scheduled visits during a 12-month period were included in the analysis. We used McNemar’s Chi-square tests to test for differences in proportions (bivariable analysis) of participants reporting risk behaviors at the baseline and the fourth visit (the month 12 visit). We used generalized estimating equations (GEE) to identify baseline participant characteristics that were associated with adopting sexual practices less likely to be associated with HIV transmission during follow-up (i.e., changing from reporting sex without a condom at baseline to reporting abstinence or 100% condom use at the first follow-up visit). This allowed us to account for correlations within subjects measured longitudinally. Variables with a p-value ≤0.10 in bivariable analysis were included in a multivariable analysis.

## Results

### Participant recruitment and characteristics

A total of 830 PLHIV agreed to enroll and completed the baseline PwP risk assessment questionnaire at the five hospitals. Of these, 756 (91.1%) completed four visits and were included in the analysis. The median age of the 756 participants was 37 years, 400 (52.9%) were female, and 438 (57.9%) had completed high school or a higher level of education. The median time since HIV diagnosis was 5 years. Participants had a median CD4 count of 356 cells/mm^3^ at study enrollment and 632 (83.6%) were taking ART (Table 2).

**Table 2:** Baseline characteristics of people living with HIV enrolled in the prevention with positives intervention assessment at five hospitals, Thailand, 2008–2009.

Characteristics (N = 756)	n (%)
**Site where participants enrolled**	
Siriraj Hospital	235 (31.1)
Vajira Hospital	199 (26.3)
Taksin Hospital	161 (21.3)
Wiang Pa Pao Hospital	84 (11.1)
Rajavithi Hospital	77 (10.2)
**Sex**	
Female	400 (52.9)
Male	356 (47.1)
**Median age in years** (range)	37 (19–72)
**Marital status**	
Single	156 (20.6)
Married or living with live-in partner	437 (57.8)
Divorced or separated	163 (21.6)
**Education**	
≤primary school	317 (41.9)
Attended or completed secondary school	328 (43.4)
Bachelor degree or higher	110 (14.6)
**Occupation**	
Irregularly employed	272 (36.0)
Unemployed	131 (17.3)
Employee	117 (15.5)
Civil servant	69 (9.1)
Others	167 (22.1)
**Self-reported source of HIV infection**	
Heterosexual sex	625 (82.7)
Blood transfusion	18 (2.4)
Injecting drugs	34 (4.5)
Homosexual sex	47 (6.2)
Unknown/other	30 (4.0)
**Median time since HIV diagnosis in years** (range)	5 (0.3–21)
**Currently on antiretroviral therapy**	632 (83.6)
**Median CD4** (cells/mm^3^) (range)	356 (1–1793)

At baseline, 427 (56.5%) participants reported having sex during the previous 3 months including 39 (9.1%) who reported sex with a casual partner and 20 (4.7%) who reported sex with a sex worker. Among participants who had sex, 338 (79.2%) reported using condoms 100% of the time (data not shown) and 89 (20.8%) reported having vaginal or anal sex without a condom during the previous 3 months (Table 3). Among the 475 PLHIV with a steady partner, 406 (85.5%) had disclosed their HIV status to their partners, and 353 (74.3%) of all the partners had been tested for HIV; 132 (37.4%) partners tested HIV negative (i.e., HIV serodiscordant). ART adherence data were available on 625 participants; 616 (98.6%) reported ≥95% adherence during the past month. Among the 427 participants who reported having sex during the previous 3 months, 403 (94.4%) reported using contraception; 355 (88.1%) used condoms and 114 (28.3%) had been sterilized. A total of 150 (19.8%) participants reported no sexual intercourse (abstinence) during the 3 months before baseline and all follow-up visits through the fourth visit.

**Table 3:** Risk behaviors reported by people living with HIV participating in the prevention with positives intervention assessment, Thailand, 2008–2009.

Strategies/Behaviors (N = 756)	Visit		P value
	Baseline (first visit)	Month 12 (fourth visit)	
	n (%)	n (%)	
**Risk behaviors during the previous 3 months**			
Had vaginal or anal sexual intercourse	427/756 (56.5)	413/756 (54.6)	0.27
Had vaginal or anal sex without a condom	89/427 (20.8)	21/413 (5.1)	<0.001*
Had sex with a casual partner	39/756 (5.2)	17/756 (2.2)	<0.001
Had sex without a condom with a casual partner	5/39 (12.8)	1/17 (5.9)	0.44*
Had sex with a sex worker	20/756 (2.6)	8/756 (1.1)	0.01
Had sex without a condom with a sex worker	6/20 (30.0)	0/8 (0.0)	N/A
Used illicit drugs^#^	11/756 (1.5)	18/756 (2.4)	0.21
Consumed ≥one alcohol drink	98/756 (13.0)	49/756 (6.5)	<0.001
Men who had sex with men	23/356 (6.5)	20/356 (5.6)	0.53
Had sex without a condom	4/23 (17.4)	2/20 (10.0)	0.67**
Was paid or received gifts for sex	9/756 (1.2)	3/756 (0.4)	0.11
Had sex without a condom	5/9 (55.6)	0/3 (0.0)	0.20**
Injected drugs	3/756 (0.4)	0/756 (0.0)	0.25
Had sex without a condom	1/2^¶^ (50.0)	0/0 (0.0)	N/A
**Diagnosis of sexually transmitted infection (STI) during the previous 3 months**			
Diagnosed with an STI^@^	4/756 (0.5)	2/756 (0.3)	0.69
Had STI symptom(s)^@@^	43/756 (5.7)	13/756 (1.7)	<0.001
**HIV disclosure to partner**			
Had a steady partner^	475/756 (62.8)	462/756 (61.1)	0.17
HIV status disclosed to steady partner	406/475 (85.5)	414/462 (89.6)	0.01
Steady partner tested for HIV	353/475 (74.3)	384/462 (83.1)	<0.001
Steady partner HIV status			
HIV positive	217/353 (61.5)	231/384 (60.1)	
HIV negative	132/353 (37.4)	150/384 (39.0)	
Unknown	4/353 (1.1)	2/384 (0.5)	
**ART adherence**^$^ **and HIV viral load**			
≥95% ART adherence	616/625 (98.6)	621/625 (99.4)	0.27
HIV viral load ≤50 copies/mL	229/349 (65.6)	487/553 (88.1)	<0.001
Median HIV viral load in copies/ml (range)	50 (0, 484000)	47 (40, 750000)	
**Pregnancy plans and contraceptive use**			
Planning a future pregnancy	26/756 (3.4)	4/756 (0.5)	<0.001
Contraceptives used (sexually active participants)	403/427 (94.4)	398/413 (96.4)	0.10
Hormonal pills	31/403 (7.7)	23/398 (5.8)	
Hormonal injection	10/403 (2.5)	7/398 (1.8)	
Hormonal implantation	21/403 (5.2)	31/398 (7.8)	
Condoms	355/403 (88.1)	372/398 (93.5)	
Condoms with others (specify)	132/355 (37.2)	157/372 (42.2)	
Condoms and oral pills	25/132 (18.9)	22/157 (14.0)	
Condoms and hormonal injection	5/132 (3.8)	7/157 (3.2)	
Condoms and hormonal implantation	15/132 (11.4)	29/157 (18.5)	
Condoms and sterilization	87/132 (65.9)	99/157 (63.1)	
Sterilization	114/403 (28.3)	116/398 (29.1)	

*Z test for proportion

** Fisher exact test was used when the observed value was less than 5 in each cell

^#^ Illicit drugs included injecting drugs (e.g., heroin) and hallucinogens.

^¶^ Two people who injected drugs had sex during the last 3 months

^@^ STI includes clinician-diagnosed genital ulcer disease; a positive test result for gonorrhea, chlamydia, or trichomonas; or a positive syphilis result (i.e., RPR titer >1:8 and a reactive TPHA test).

^@@^ STI symptoms defined as having: dysuria, pain in genital area, swollen testis, vaginal discharge, pain in lower abdomen, genital ulcer, vesicles, urethral discharge, anal discharge, or wart.

^A “steady partner” was defined as a person that the participant had known for more than two months, regularly had sex with, and with whom the participant felt a psychological connection.

^$^ART adherence was assessed each visit using PLHIV self-report of antiretroviral use during the previous 3 days and the previous month and was available on 625 participants.

### PwP interventions and HIV risk behaviors

Among participants who had sexual intercourse, the proportion of participants reporting sex without a condom decreased from 20.8% (89 of 427) at baseline to 5.1% (21 of 413) at month 12 (p<0.001) and the proportion reporting sex without a condom with a casual partner decreased from 12.8% (5 of 39) at baseline to 5.9% (1 of 17) at month 12 (p = 0.44) (Table 3). Of the 356 male participants, 23 (6.5%) reported having sex with a man during the 3 months before enrollment and four (17.4%) did not use a condom; 20 (5.6%) reported sex with a man during the 3 months before the month 12 visit and two (10%) did not use a condom (P = 0.67). Only nine (1.2%) participants reported that they had sex in exchange for money or gifts, and three (0.4%) reported injecting drugs at baseline. About half of the participants who reported having sex for money and who reported injecting drugs at baseline had sex without using a condom. None of the three participants who reported having sex in exchange for money at month 12 reported they had sex without a condom, but the change was not statistically significant.

Among the 475 participants with a steady partner, 406 (85.5%) reported that they had disclosed their HIV status to their partners at baseline; at the month 12 visit, 414 (89.6%) of the 462 participants with steady partners had disclosed their HIV status (p = 0.01) and partner HIV testing increased from 74.3% (353 of 475 partners) at baseline to 83.1% (384 of 462 partners) at the month 12 visit (p<0.001). Fewer participants reported they were planning a future pregnancy at the month 12 visit than at baseline (p<0.001). The proportion of participants with an HIV viral load ≤50 copies/mL increase from 229 (65.6%) of 349 participants at baseline to 487 (88.1%) of 553 participants at the month 12 visit (p<0.001).

### Factors associated with abstinence and condom use

The number of participants who reported that they had abstained from sex or used condoms 100% of the time during the previous 3 months (i.e., participants who did not report having vaginal or anal sex without a condom in Table 3) increased from 667 (88.2%) at baseline to 735 (97.2%) at the month 12 visit (adjusted odds ratio [aOR] 1.9, 95% confidence interval [CI] 1.6 to 2.5, p<0.001, per visit) (data not shown). Factors associated with an increase in sexual practices less likely to be associated with HIV transmission (i.e., abstaining from sex or 100% condom use) in multivariable analysis included: having sought care at Vajira (p<0.001), Taksin (p<0.001) or Wiang Pa Pao (p = 0.02) Hospitals compared with Siriraj Hospital; having been diagnosed with HIV for 3 or more months, compared with less than 3 months (p<0.001); not receiving condoms at the first visit, compared with having received condoms (p = 0.007); and having received risk reduction messages from a doctor, compared with having received risk reduction messages from a health care provider that was not a doctor (e.g., a nurse or counsellor) (p = 0.002) (Table 4). Based on the results of the GEE analysis, participants in these groups were about 50% less likely to engage in sexual behaviors associated with HIV transmission during follow-up than reported at baseline.

**Table 4:** Results of generalized estimating equation analysis of baseline factors associated with change from condomless sex to abstinence or 100% condom use at follow-up visits among PLHIV participating in prevention with positives intervention assessment, Thailand, 2008–2009.

Variables	Bivariate analysis		Multivariate analysis	
	OR*(95% CI)	p-value	aOR*(95% CI)	p-value
**Clinical visit** (n = 756)				
Fourth	4.7 (3.0–7.3)	<0.001	4.8 (3.0–7.5)	<0.001
Third	6.1 (3.7–10.3)	<0.001	6.1 (3.6–10.5)	<0.001
Second	2.4 (1.7–3.2)	<0.001	2.4 (1.7–3.3)	<0.001
First	1.0		1.0	
**Hospital** (n = 756)				
BMA Vajira Hospital	4.1 (2.2–7.8)	<0.001	3.5 (1.8–6.7)	<0.001
BMA Taksin Hospital	3.1 (1.8–5.5)	<0.001	2.8 (1.6–4.9)	<0.001
Wiang Pa Pao Hospital	2.4 (1.2–5.1)	0.20	2.5 (1.4–5.4)	0.02
Rajavithi Hospital	0.9 (0.5–1.5)	0.61	1.0 (0.5–1.8)	0.98
Siriraj Hospital	1.0		1.0	
**Received condoms at first visit** (n = 756)				
Yes	0.5 (0.3–0.7)	<0.001	0.5 (0.3–0.8)	0.007
No	1.0		1.0	
**Received short risk reduction messages from doctor at baseline** (n = 756)				
Yes	1.7 (1.1–2.4)	0.002	1.9 (1.2–2.8)	0.002
No	1.0		1.0	
**Disclosure to steady partner prior to first visit** (n = 756)				
Yes (or no steady partner)	1.5 (1.0–2.3)	0.03	NS	
No	1.0		1.0	
**Steady partner tested for HIV prior to first visit** (n = 756)				
Yes (or no steady partner)	1.4 (0.9–2.0)	0.10	NS	
No/don’t know	1.0		1.0	
**At first visit, time since HIV diagnosis** (n = 746)				
<3 months	0.4 (0.2–0.6)	<0.001	0.4 (0.2–0.6)	<0.001
≥3 months	1.0		1.0	
**At first visit, alcohol or drug use during previous 3 months** (n = 756)				
Yes	0.6 (0.4–0.9)	0.02	NS	
No	1.0		1.0	

OR odds ratio, aOR adjusted odds ratio, CI confidence interval

* Adjusted for visit number

## Discussion

We developed and implemented a PwP program in hospitals in Thailand. After participating in the PwP program for one year, PLHIV were more likely to have disclosed their HIV status to their partner, to have used condoms when having vaginal or anal sex, and to have an HIV viral load result ≤50 copies/mL than they were at baseline. This is consistent with previous studies [12, 19–21] that have shown that PwP interventions delivered to groups or individuals can effectively reduce risk behaviors associated with HIV transmission.

PLHIV who had been diagnosed with HIV for 3 or more months were more likely to report abstinence or 100% condom use than PLHIV who had been diagnosed for less than 3 months and PLHIV were more likely to report abstinence or 100% condom use at follow-up visits than at baseline. Other PwP assessments have also shown that participants’ sexual risk behaviors decrease over time [22] suggesting that a longer participant-health care provider relationship and greater exposure to PwP messages may be associated with an increased uptake of PwP activities [10]. PLHIV who received risk reduction messages from a doctor were more likely to report abstinence or 100% condom use during follow-up than participants who received these messages from other staff. This is consistent with other studies that have shown that doctors can have an important impact on patient behaviors [23].

Our study also found that PLHIV who received care at Vajira and Taksin Hospitals, managed by the city government of Bangkok (i.e., BMA), were more likely to report abstinence or 100% condom use during follow-up than PLHIV who received care at the University Hospitals in Bangkok. HIV clinic staff at the BMA hospitals provide PwP services as part of routine clinic activities and have established long-term relationships with clinic patients and other hospital staff. A case manager was hired to provide PwP services at Siriraj Hospital for this project. The Siriraj Hospital PwP staff may not have had sufficient time to develop strong relationships with patients to support behavior change. In addition, Siriraj Hospital is a national referral center and likely cares for a higher proportion of PLHIV with a history of treatment failure, adherence challenges, and higher HIV associated risk behavior than PLHIV at other hospitals.

PLHIV who did not receive condoms at their first visit were more likely to report abstinence or 100% condom use during follow-up than PLHIV who received condoms. This finding is counter-intuitive. However, condoms were not randomly distributed during the study; health care providers gave condoms to PLHIV who they perceived were sexually active or at risk of HIV or STI transmission and to PLHIV requesting condoms (Fig 1). This may have resulted in a selection bias with PLHIV who received condoms representing a population at a higher risk of having sexual intercourse without a condom than those who did not receive condoms. It may also indicate that those who engaged in risk behaviors did not receive a sufficient number of condoms or that distributing condoms alone is not an adequate prevention strategy for this group. Further investigation to understand why these patients continued to engage in sexual activities associated with HIV infection is necessary.

During the study, self-reported ART adherence was high (i.e., 99%) and the proportion of PLHIV with HIV viral load results ≤50 copies/mL at the fourth visit was higher than baseline. Nonetheless, although PLHIV in this study reported high levels of ART adherence, 12% had a detectable viral load at the month 12 visit, suggesting that self-reported adherence may not reflect true adherence or that HIV drug resistance may exist among some participants [24]. We found that almost 40% of participants were in HIV serodiscordant relationships which is in line with other reports [25]. The number of PLHIV in serodiscordant relationships and the frequency that virus is detectable among PLHIV receiving ART highlights the importance of PwP services and routine viral load monitoring to identify PLHIV with adherence challenges or ART resistance who may need additional adherence support or a change in their ART regimen. Antiretroviral pre-exposure prophylaxis (PrEP) can reduce the risk of HIV transmission to HIV-uninfected partners in serodiscordant relationships [26] and the 2014 Thailand National HIV Treatment and Care Guidelines recommend that providers consider PrEP for HIV-uninfected partners who do not use condoms consistently as part of a comprehensive HIV prevention strategy [1].

In Thailand, patients are usually assessed for STIs only when they report symptoms to their care providers [27]; however, in the PwP program, participants were asked about STI symptoms at each visit and participants with symptoms were treated. We found a decrease in STI symptoms and diagnoses at the month 12 visit compared to baseline, consistent with studies [28] showing that implementing interventions to diagnose and treat STIs among PLHIV can reduce STI incidence.

Several studies have shown that PwP interventions can increase the proportion of PLHIV who disclose their HIV status to their partners [21, 25]. In this study, we found that a relatively high proportion of PLHIV (i.e., 86%) had disclosed their HIV status to their partners at the baseline visit, and that many of their partners had been tested for HIV. This is a higher level of partner HIV testing than reported in other locations [25, 29–31] and may be because many PLHIV in our PwP program had been receiving HIV care and treatment services for several years, and HIV services, including testing, are widely accessible free of charge in Thailand. Unlike other PwP evaluations [32–34], we did not find an increase in contraceptive use among sexually active PLHIV during follow-up. The high level of contraceptive use at baseline (i.e., 94%) may have made additional gains challenging [8].

This assessment of the implementation of a PwP program has several limitations. The assessment was focused in hospitals where PwP interventions were implemented and we did not have a control group. Data used in the analysis were collected in 2008–2009 and may not reflect the current situation in Thailand [14, 31]. Our analysis was limited to data collected from PLHIV evaluated at tertiary care facilities in Bangkok and a community hospital in Chiang Rai, results from other settings may differ. We used participant self-reports of risk behaviors that are vulnerable to social desirability bias and may result in under-reporting of risk behaviors [35]. We did not perform a standardized set of laboratory tests to diagnosis STIs among all participants; testing was based on clinical signs and symptoms. This may have led to an underestimate of STI prevalence among PLHIV. Our analysis was limited to a highly selected population of participants who successfully completed a baseline assessment and three follow-up visits. This may have selected for participants more likely to adhere to clinical recommendations. In addition, most participants in this study were heterosexual and did not inject drugs. The effect of this PwP package may differ among people who inject drugs and MSM and additional research is clearly needed.

In summary, our analysis of PwP services at five hospitals in Thailand showed that the implementation of a PwP program was associated with a decrease in risk behaviors that can lead to HIV transmission. Based on these findings, the Thailand MOPH began to scale-up the PwP model throughout the country in 2010–2011 [36] and the model has been integrated into routine HIV care. The capacity to deliver HIV PwP services should be strengthened at hospitals delivering HIV treatment and care services. Further studies are needed to assess the long-term impact of PwP interventions on HIV incidence in the era of “test and treat regardless of CD4 count” and the impact of PwP interventions on key populations including MSM, transgender people, and people who inject drugs.
